# EGFR-Mutant Lung Adenocarcinoma Mimicking a Pneumonia

**DOI:** 10.1155/2012/257827

**Published:** 2012-08-23

**Authors:** Álvaro Taus, Flavio Zuccarino, Carlos Trampal, Edurne Arriola

**Affiliations:** ^1^Medical Oncology Department, Hospital del Mar-Parc de Salut Mar, Passeig Maritim 25-29, 08003 Barcelona, Spain; ^2^Radiology Department, IDIMAS-CRC, Hospital del Mar, Passeig Maritim 25-29, 08003 Barcelona, Spain; ^3^PET Unit Molecular Imaging Center, CRC CIM-CRC Mar, Passeig Maritim 25-29, 08003 Barcelona, Spain

## Abstract

PET-CT scan has demonstrated to be very effective in lung cancer diagnosis and staging, but lung cancer has multiple ways of presentation, which can lead to an error in diagnosis imaging and a delay on the beginning of specific treatment. We present a case of a 77-year-old man with an initial PET-CT scan showing high 18F-FDG intake, suggesting a bilateral pneumonia, who was finally diagnosed of an EGFR-mutant lung adenocarcinoma. EGFR-activating mutation allowed us to start treatment with the oral tyrosin kinase inhibitor Gefitinib, obtaining a rapid and sustained response. Histological confirmation of imaging findings is always necessary to avoid diagnostic errors.

## 1. Introduction

Staging of nonsmall-cell lung cancer was one of the first approved indications for the use of positron emission tomography (PET) [1]. Since 2001, combined PET and computed tomography (PET-CT scan) has rapidly replaced stand-alone PET and has become a key tool in the staging of lung cancer [2]. Although [18F] Fluoro-2-deoxy-D-glucose (18F-FDG) has high sensitivity for cancerous conditions, there are benign processes that result in abnormal accumulation of and false positive images. These false positive results are due to conditions where 18F-FDG accumulation occurs in metabolically active tissue that is not cancerous, such as infection or inflammatory processes [3].

## 2. Case Report

We present a 77-year-old man, with no history of smoking, admitted to the emergency room with a 2 month history of malaise, shortness of breath, and weight loss. His medical history involved controlled heart failure, arterial hypertension, hypercholesterolemia, and obstructive sleep apnea syndrome. Blood count, liver, and renal functions were normal. Chest X-ray showed areas of consolidation in both lung bases, predominantly left.

**Figure 1:**
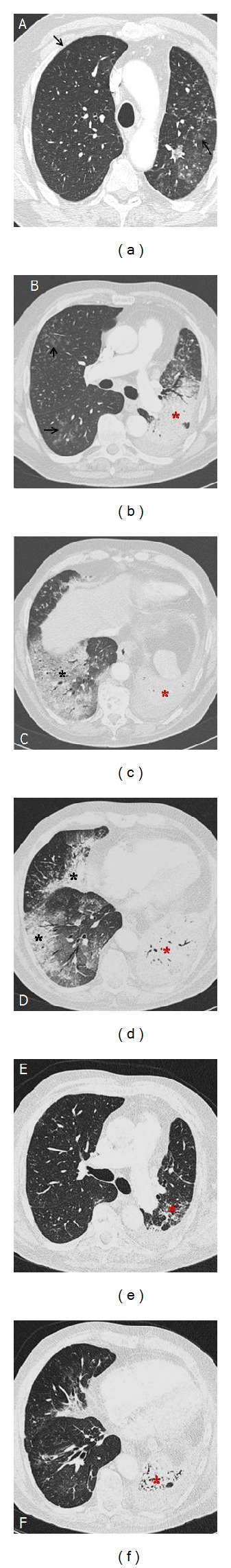
CT-scan of the chest shows bilateral nonsolid pulmonary nodules (a and b: *black arrows*) and ill-defined areas of pulmonary opacities with “crazy-paving” pattern in right lower and middle lobes (c, d: *black asterisk*). In left lung extensive air-space consolidation is present (b, c, and d: *red asterisk*). Follow-up CT-scan realized 8 months later after Gefitinib treatment shows no evidence of nonsolid pulmonary nodules and of right pulmonary opacities with an important reduction of left consolidation (e, f; *red asterisk*).

A CT-scan of the chest demonstrated diffuse bilateral ground glass nodules, ill-defined areas of pulmonary opacities with “crazy-paving” pattern in right lower and middle lobes, and extensive air-space consolidation in left lung (Figures 1(a), 1(b), 1(c), and 1(d)). These findings suggested inflammatory or infectious process as first choice, being less likely neoplasic aetiology or organizing pneumonia.

**Figure 2:**
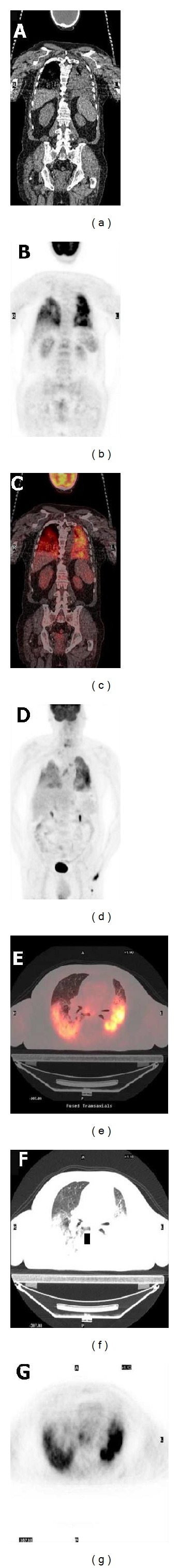
PET-CT scan shows extensive and heterogeneous 18F-FDG uptake in both lungs in correlation with bilateral ground glass images and bilateral ill-defined pulmonary opacities on CT image (coronal planes (a, b, and c), volumetric projection (d), and axial planes (e, f, and g)). Hypermetabolic lymph nodes are observed in right supraclavicular, left mediastinal, and subcarinal regions (volumetric projection (d)) as well as subcarinal hypermetabolic nodes (axial planes (e and g)).

The PET-CT scan (low-dose CT) reported an extensive and heterogeneous deposit of [18F] Fluoro-2-deoxy-D-glucose (18F-FDG) in both lungs with a maximum standardised uptake value (SUV) of 11.30, that correlated with morphological findings described on CT scan. In addition, hypermetabolic lymph nodes were detected in right supraclavicular, left mediastinal, and subcarinal regions (maximum SUV of 6,06). These PET-CT findings suggested as first choice a bilateral inflammatory or infectious process (Figure 2).

Bronchoscopy demonstrated serous secretions predominantly in the left bronchial tree. Bronchial aspirate, bronchoalveolar lavage, and bronchial biopsy resulted positive for adenocarcinoma. All bacteriological tests performed were negative.

In this case an activating mutation on exon 19 of epidermal growth factor receptor (*EGFR*) gene was found. Activating *EGFR* mutations derive in increase of response to EGFR tyrosin kinase inhibitors when comparing with standard chemotherapy [4, 5]. This finding allowed us to start treatment with the EGFR oral tyrosin kinase inhibitor Gefitinib. Rapid and sustained response was observed in the follow-up CT scan, and the patient remains on Gefitinib for 8 months without evidence of progression (Figures 1(e) and 1(f)) and excellent tolerance.

## 3. Discussion

Lung cancer has multiple ways of presentation, which can lead to an error in diagnostic imaging, therefore histological confirmation is always necessary. Because EGFR-mutant tumours show lower 18F-FDG uptake in PET-CT scan [6], this case illustrates a rare presentation of *EGFR*-mutant lung adenocarcinoma with high 18F-FDG mutant lung adenocarcinoma.

In this case, with initial imaging/metabolic procedures suggesting bilateral inflammatory or infectious process, delay of histological confirmation would have had a negative impact in patient's survival and quality of life.
